# Estimation of Montreal Cognitive Assessment Scores Using Caregiver Reports and Demographics: A Model Development Study

**DOI:** 10.3390/jcm15134945

**Published:** 2026-06-25

**Authors:** Jungmin So, Moon-Ho Park

**Affiliations:** Department of Neurology, Korea University Ansan Hospital, Ansan 15355, Republic of Korea; wjdalsth1029@gmail.com

**Keywords:** MoCA, caregiver, estimation

## Abstract

**Background/Objectives**: Assessment of cognitive function in patients with dementia is often hindered by functional and environmental barriers. Although caregiver reports are an alternative source, their clinical utility for estimating patients’ cognitive function remains uncertain. This study aimed to estimate cognitive function using caregiver-reported data combined with patient demographics and to evaluate its clinical utility. **Methods**: This retrospective cross-sectional study enrolled participants who visited a memory clinic and completed the Montreal Cognitive Assessment (MoCA) for cognitive assessment, together with caregiver-reported questionnaires for activities of daily living (ADL) and neuropsychiatric symptoms (NPS). Multivariable linear regression models were constructed to predict the MoCA score, with Model 1 including demographics, ADL, and NPS as covariates and Model 2 further incorporating clinical diagnosis. The intraclass correlation coefficient, Bland–Altman analysis, and regression error characteristic curves were assessed. **Results**: Among 2650 participants (56.5% women; mean age, 70.4 years), the NPS variable was excluded from both models. Model 1, which included demographics and ADL, explained 65.4% of the variance, whereas Model 2, which incorporated clinical diagnosis, explained 75.9%. Model 2 yielded an intraclass correlation coefficient of 0.853, compared to 0.778 for Model 1. At a 4-point error tolerance, Model 2 yielded an accuracy of 75.5%. Bland–Altman biases were near zero, with 95% limits of agreement of approximately ±7 points for Model 2. **Conclusions**: MoCA scores can be estimated using caregiver-reported ADL scores, demographics, and clinical diagnosis. NPS scores provided no additional predictive value when these factors were included. These models provide valid quantitative tools for indirect cognitive assessment when in-person testing is impossible.

## 1. Introduction

While face-to-face evaluation is the standard clinical examination, patients with dementia often encounter barriers to attending clinic visits, such as declining physical mobility, transportation difficulties, and limited support [1,2,3]. Moreover, caregivers actively manage patients’ complex daily activities and serve as the primary point of contact for physicians [4,5]. If patient visits are impossible, physicians rely almost entirely on proxy reports from caregivers to track the patients’ functional changes, behavioral symptoms, and overall disease progression [6]. The importance of this caregiver-provided information was further highlighted when remote care delivery was required, such as during the COVID-19 pandemic [7].

The association between caregiver-reported functional status and patients’ cognitive performance is well established in neuropsychology [8,9,10,11]. Informant-based measures of functional and cognitive decline have been shown to correlate significantly with both objective neuropsychological performance and neurobiological markers of Alzheimer’s disease [10,11,12]. Previous studies have investigated the usefulness of informant-based questionnaires, including the Informant Questionnaire on Cognitive Decline in the Elderly (IQCODE) [8] and the Eight-item Interview to Differentiate Aging and Dementia (AD8) [9], for identifying cognitive impairment, showing that these instruments can effectively distinguish cognitively normal individuals from those with mild cognitive impairment or dementia. Despite the increasing need for caregiver reports in clinical practice, it remains unclear whether cognitive screening can be meaningfully estimated from unverified functional and behavioral measures. The Montreal Cognitive Assessment (MoCA) is a validated instrument for screening cognitive impairment and tracking the trajectory of dementia progression [13,14]. In circumstances where direct administration of the MoCA is not feasible, score estimation based on caregiver-reported data can support clinicians in maintaining care continuity and guiding informed clinical decision-making.

Therefore, this study aimed to determine whether a patient’s MoCA score can be reliably estimated using demographic variables combined with caregiver-reported activities of daily living (ADL) and neuropsychiatric symptoms (NPS) evaluations. We also assessed the accuracy and reliability of the estimates to determine their clinical utility.

## 2. Materials and Methods

### 2.1. Study Design and Participants

This retrospective cross-sectional study was conducted at the Memory Clinic of Korea University Ansan Hospital. We included 2650 consecutive participants who visited the clinic between January 2020 and December 2025 and met the following criteria: (1) both the patient and caregiver attended the clinic; (2) the patient completed the MoCA assessment; and (3) the caregiver completed both the ADL and NPS questionnaires. All participants underwent a clinical diagnostic protocol that included routine blood tests, neuropsychological testing, and brain MRI primarily to exclude the possibility of medical conditions and structural brain lesions that might influence cognitive function, such as acute stroke, brain tumors, hydrocephalus, and traumatic brain injury. This study was conducted in accordance with the guidelines of the Declaration of Helsinki. This study was approved by the Institutional Review Board of the Korea University Ansan Hospital (IRB No. 2026AS0131). Informed consent was not required because of the retrospective nature of the study.

### 2.2. Clinical Assessments

Demographic data, including age, sex, and years of education, were collected. Patients were administered the MoCA, a 30-point screening tool that assesses short-term memory, visuospatial abilities, executive function, attention, language, and orientation [13].

The caregivers completed questionnaires to assess the patients’ functional abilities and behavioral symptoms. Functional abilities were measured using the Seoul-Instrumental ADL, a culturally adapted Korean scale that evaluates complex daily tasks required for independent living, such as managing finances, shopping, preparing meals, and managing medications [15]. The scores ranged from 0 to 45, with higher scores indicating greater functional impairment. NPS was measured using the Neuropsychiatric Inventory (NPI), a validated caregiver interview covering 12 domains: delusions, hallucinations, agitation/aggression, depression/dysphoria, anxiety, elation/euphoria, apathy/indifference, disinhibition, irritability/lability, aberrant motor behavior, nighttime behavioral disturbances, and appetite/eating abnormalities [16]. The total NPI scores range from 0 to 144, with higher scores indicating a greater NPS burden.

Clinical diagnosis was established by neurologists based on standardized criteria. Cognitively normal (CN) participants performed normally in all test domains of the neuropsychological test battery [17]. Petersen criteria were used to diagnose mild cognitive impairment (MCI) [18]. Patients with dementia fulfilled the criteria for major neurocognitive disorders proposed in the Diagnostic and Statistical Manual of Mental Disorders, Fifth Edition [19].

### 2.3. Statistical Analysis

Descriptive data are presented as means and standard deviations (SDs) for continuous variables and frequencies and percentages for categorical variables. The dataset was randomly divided into training (60%, *n* = 1590) and test (40%, *n* = 1060) data.

Two multivariate linear regression models with stepwise selection were developed using the training data, with the MoCA score as the dependent variable. Model 1 included demographic data, ADL scores, and NPS scores as independent variables, whereas model 2 included clinical diagnosis as an additional variable. These models were validated using root mean square error (*RMSE*), coefficient of determination (*R*^2^), and mean absolute error (*MAE*) [20]. For clinical utility, the two models were visualized as nomograms using the ordinary least squares method [21,22]. Each variable was assigned a point value, and summing these points yielded a total score that directly mapped to the predicted (estimated) MoCA scores in these nomograms.

Agreement between the predicted and observed MoCA scores was assessed using test data using the intraclass correlation coefficient (ICC) and Bland–Altman analysis [23,24]. The ICC was calculated using a single-measure, two-way, mixed-effects model for absolute agreement. ICC values were interpreted as indicating poor (<0.50), moderate (0.50–0.75), good (0.75–0.90), or excellent (>0.90) agreement. For the Bland–Altman analysis, the mean difference (bias) and 95% limits of agreement (mean difference ± 1.96 SDs) were presented. A mean difference close to zero indicated a higher degree of agreement between the predicted and observed MoCA scores, whereas narrow limits of agreement indicated high predictive precision.

To evaluate the overall reliability and accuracy of the model, we analyzed the heteroscedasticity of the prediction errors, generated calibration plots, and constructed regression error characteristic (REC) curves. Heteroscedasticity was assessed by calculating the Pearson correlation coefficient (*r*) between the absolute residuals and predicted MoCA scores [25]. The absolute correlation coefficient (|*r*|) was interpreted as indicating a small (0.10–0.29), medium (0.30–0.49), or large (≥0.50) relationship [26]. The calibration of each model was visually assessed using scatter plots of the predicted versus observed MoCA scores. A locally estimated scatterplot smoothing (LOESS) curve with bootstrapped 95% confidence intervals (CIs) is presented with a 45-degree reference line to represent perfect concordance [27].

REC curves compare the absolute difference between the predicted and observed MoCA scores with the cumulative proportion within that tolerance [28]. The overall performance was evaluated using the area under the REC curve (AUC-REC) with 95% CIs using a bootstrapping procedure with 1000 replicates. A 4-point error tolerance was defined as the threshold for acceptable predictive accuracy. This threshold was based on the established minimum detectable change and reliable change indices for the MoCA, which conventionally fall within the range of 4 to 5 points [29,30,31].

Statistical significance was set at a two-sided *p* < 0.05. All statistical analyses were performed using R version 4.4.3 (R Foundation for Statistical Computing, Vienna, Austria).

## 3. Results

### 3.1. Baseline Characteristics

Among the 2650 participants (age, 70.41 ± 10.97 years; 43.5% male; education, 8.60 ± 4.65 years), the mean scores were 15.75 ± 6.79 on the MoCA, 12.42 ± 12.29 on the ADL, and 3.88 ± 7.37 on the NPS. There were 307 (11.6%) CN participants, 1683 (63.5%) with MCI, and 660 (24.9%) with dementia.

### 3.2. Model Development

In Model 1, which did not include clinical diagnosis as a covariate, stepwise variable selection retained four predictors but excluded the NPS score. All four retained predictors were statistically significant, and the ADL score was the strongest predictor (*β* = −0.334, *t* = −37.98), followed by education (*β* = 0.523, *t* = 20.48), age (*β* = −0.071, *t* = −6.80), and sex (*β* = 1.121, *t* = 5.14) (all *p* < 0.001). The model explained 65.4% of the variance in the observed MoCA scores (*F* = 748.3, *p* < 0.001), with a residual standard error of 4.08.

In Model 2, which incorporated clinical diagnosis, stepwise selection retained five predictors, excluding the NPS score. All five predictors were statistically significant, and clinical diagnosis was a strong independent predictor (*β* = −5.116, *t* = −26.31), followed by education (*β* = 0.504, *t* = 23.64), ADL score (*β* = −0.174, *t* = −18.35), age (*β* = −0.062, t = −7.16), and sex (*β* = 0.918, *t* = 5.04) (all *p* < 0.001). The model explained 75.9% of the variance (*F* = 998.3, *p* < 0.001), with a residual standard error of 3.41.

### 3.3. Validation and Agreement

**Figure 1:**
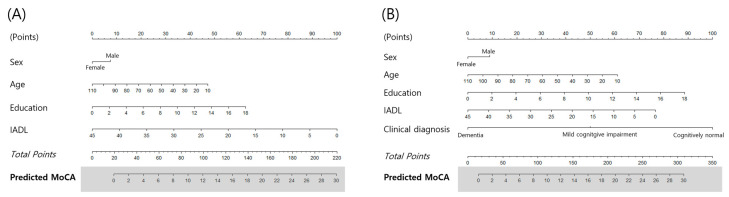
Nomogram for predicting Montreal Cognitive Assessment (MoCA) Scores. (**A**) Model excluding clinical diagnosis; (**B**) Model including clinical diagnosis. To use the nomogram, locate the patient’s value for each variable on its corresponding axis and assign the points shown on the top “Points” scale. The points from all the variables are summed to find the “Total Points.” Finally, the predicted MoCA score (range: 0–30) on the lowest axis is directly below this total.

For the internal validation, Model 1 showed an *RMSE* of 4.07, *R*^2^ of 0.617, and *MAE* of 3.21. Model 2 showed an *RMSE* of 3.42, *R*^2^ of 0.730, and *MAE* of 2.69. For clinical utility, Figure 1 presents the nomograms for both models.

**Figure 2:**
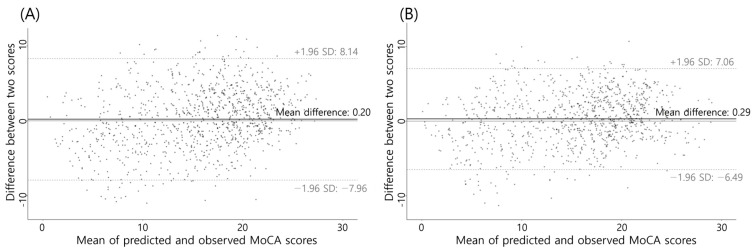
Bland–Altman plots for predicted versus observed MoCA. (**A**) Model excluding clinical diagnosis; (**B**) Model including clinical diagnosis. The solid centerline indicates the mean difference, and the dashed outer lines represent the 95% limits of agreement.

For the agreement between the predicted and observed MoCA scores, the ICC values were 0.778 for Model 1 and 0.853 for Model 2. Bland–Altman plots show this relationship (Figure 2). The groups showed a mean difference near zero. Model 2 showed slightly narrower 95% limits of agreement at approximately ±7 points compared with ±8 points for Model 1.

**Figure 3:**
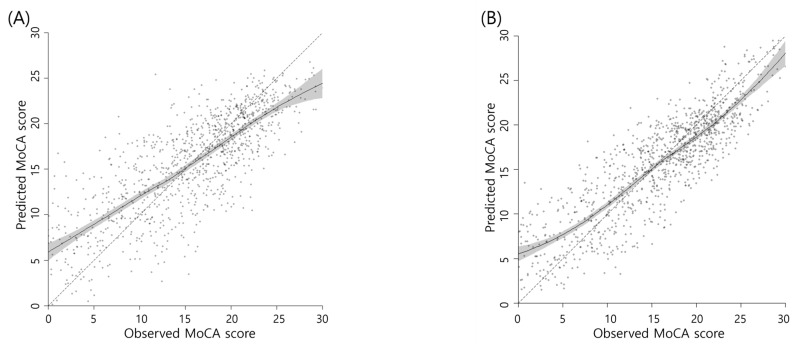
Calibration plot of predicted versus observed MoCA scores. (**A**) Model excluding clinical diagnosis; (**B**) Model including clinical diagnosis. The x-axis represents the observed MoCA scores, and the y-axis represents the predicted MoCA scores. The dashed line indicates ideal calibration, where the predicted and observed scores are identical (y = x). The solid black line illustrates the model trend fitted via the locally estimated scatterplot smoothing (LOESS) method, with the shaded gray area indicating the 95% CIs.

For heteroscedasticity, the correlation coefficients were 0.158 (*p* < 0.001) for Model 1 and 0.144 (*p* < 0.001) for Model 2. Overall heteroscedasticity remained small in both models. Calibration plots showed agreement between the predicted and observed scores (Figure 3). Model 2 demonstrates a closer alignment than Model 1, particularly at the extremes of the score distribution.

**Figure 4:**
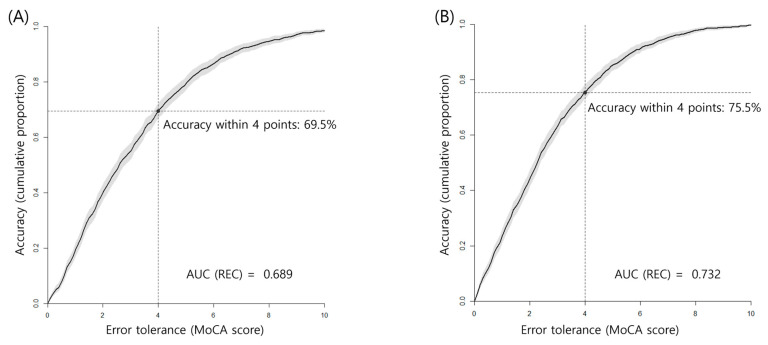
Regression Error Characteristic (REC) curves for the MoCA score prediction models. (**A**) Model excluding clinical diagnosis; (**B**) Model including clinical diagnosis. The REC curves illustrate the predictive precision of the test data. The x-axis denotes error tolerance (the absolute difference between the predicted and observed scores), and the y-axis represents accuracy (the cumulative proportion of predictions within that tolerance). The solid black line shows the REC curve, and the shaded gray area indicates the 95% confidence intervals (CIs) derived from 1000 bootstrap iterations.

The REC curves are shown in Figure 4. The accuracies were 69.5% for Model 1 (AUC-REC = 0.689) and 75.5% for Model 2 (AUC-REC = 0.732).

## 4. Discussion

This study proposed two approaches to estimate patients’ MoCA scores from caregiver-reported ADL and demographics. When clinical diagnosis was incorporated, the accuracy of the estimation improved.

The clinical rationale for this study arose from a common situation in which patients with dementia frequently face barriers to clinic visits [32,33]. Although direct cognitive testing remains the standard, real-world practice requires alternative approaches in the absence of direct patient assessments. Caregivers can provide reliable information about a patient’s cognitive function and decline from premorbid levels [34,35,36]. This study extends this concept by translating caregiver-reported data into a quantitative estimate of a patient’s cognitive status. The nomograms developed here also serve as practical and accessible tools that clinicians can readily apply at the point of care.

These models can help maintain continuity of care for patients with dementia. Because not only mobility impairment and limited access to clinics, but also functional decline and behavioral symptoms often prevent patients from attending regular clinic visits, estimating cognitive status allows for continuous monitoring and timely intervention [2]. By integrating caregiver-reported data into patients’ cognitive evaluations, this approach parallels the essential role of family informants [4,5] and serves as a practical tool for remote clinical monitoring.

These two models serve distinct but complementary roles in clinical practice, each addressing different situations commonly encountered in dementia care. Model 1 excludes clinical diagnosis and is intended for initial remote cognitive estimation when diagnostic information is unavailable. Model 2 includes clinical diagnosis as an additional variable and is designed for longitudinal monitoring of patients with previously established diagnoses who cannot attend follow-up clinic visits.

In Model 1, the ADL score was the strongest predictor of MoCA performance, followed by education, age, and sex. Together, these four variables account for 65.4% of the variance. This is a substantial improvement over previous studies that demonstrated that demographics alone could explain only 24–28% of the variance [37,38]. Given the association between functional impairment and cognitive decline [39], combining ADL scores with demographics can enhance the estimation of patients’ MoCA scores. As anticipated, the addition of a clinical diagnosis to Model 2 was associated with improved overall predictive performance. Diagnosis is an important independent predictor of cognitive status. However, the utility of Model 1 remains high and therefore has practical value. It provides a useful tool for remote appointments or when a formal diagnostic classification is not yet available.

The NPS score was excluded from both final models using stepwise selection. This suggests that while NPS is clinically relevant, it cannot independently predict MoCA scores when functional assessments are accounted for. While NPS is prevalent across cognitive dysfunctions, its association with MoCA scores seems to be more complicated and nonlinear [40,41]. ADL may be related to the functional part of the NPS [42]. Thus, when caregiver-reported ADL scores and demographics are included, adding NPS data may offer no further predictive value.

Overall, the validation and agreement substantiate the clinical utility of our models. The ICC indicated good agreement between the predicted and observed MoCA scores. Although the Bland–Altman plots confirmed this tendency, the 95% limits of agreement revealed that individual predictions could still differ substantially. These findings are in line with those of a previous study that showed that correlations may obscure individual variations in cognitive testing [24]. Therefore, our model results should be considered practical approximations in clinical practice, rather than precise numerical scores.

The correlation coefficient between the absolute residuals and the predicted values yielded a small degree of heteroscedasticity. In particular, prediction errors were slightly larger at lower MoCA scores, where monitoring is most needed for patients with moderate to severe dementia. Clinically, this is plausible because patients with advanced dementia have increased heterogeneity in functional and cognitive features. Although heteroscedasticity remains small and the correlation assumption holds, the predictive accuracy may decline slightly in severe cases [43].

The calibration plots indicated that the overall agreement with the LOESS curves was close to the optimal 45-degree reference line throughout the entire MoCA range; however, a small deviation was observed at the lower end. An analysis of the REC curve was used to evaluate the predictive accuracy. Both models attained accuracies ranging from 69.5% to 75.5% at a 4-point threshold on the MoCA. This finding indicates that our models yield clinically useful predictions from caregiver-reported data and demographics.

Although the models yielded adequate accuracy at the cohort level, these may mask important limitations for single-patient prediction. Because the ±7 to 8-point margin for the 95% limits of agreement effectively stretches across the majority of the diagnostic MoCA range, any single prediction risks significant deviation from the empirically observed score. The 4-point error tolerance was exceeded in approximately one-quarter to one-third of predictions.

This study has some limitations. As a retrospective, single-center study conducted in a Korean memory clinic with relatively low mean education and a small sample size, external validation is needed to confirm generalizability across different healthcare settings, cultural contexts, and populations with higher educational attainment. All participants in this cohort attended the clinic and completed the MoCA, whereas the intended application of the models is for patients who are unable to undergo direct cognitive assessment. This discrepancy may limit generalizability, as individuals who cannot attend clinic visits may differ systematically from those included in model development with respect to disease severity, comorbidity burden, and available social support. Stepwise variable selection may produce overly optimistic performance estimates and unstable coefficients. Our validation used a single train-test split rather than cross-validation, representing internal testing rather than true external validation, and performance may be overestimated in independent samples. Although the predicted MoCA score was within the tolerable range, individual-level variations should be considered. Clinicians should cautiously use these models for adjunctive assessments. This study did not evaluate specific dementia subtypes, such as Alzheimer’s disease or vascular cognitive impairment, nor did it include various comorbidities. Clinical diagnosis was coded as an ordinal three-level variable, assuming equal intervals between diagnostic categories. The exclusion of NPS data may have reduced the predictive accuracy for patients with prominent NPS. Model 2 was developed using concurrent clinical diagnosis and MoCA scores from the same visit, whereas the intended application involves using a previously established diagnosis to estimate current cognitive status. The model’s performance when diagnosis and MoCA estimation are temporally separated remains unvalidated and requires prospective evaluation. Finally, the use of a culturally adapted ADL tool limits the direct application of the regression coefficients to other cultural populations. However, the underlying principle of estimating patients’ cognitive status using caregiver-reported functional data remains highly applicable across diverse clinical settings.

## 5. Conclusions

This study presents validated models that can predict patients’ MoCA scores using caregiver-reported ADL data and demographics. These findings support clinical continuity and facilitate the remote monitoring of patients who are unable to attend clinic visits. Although direct cognitive assessment remains the standard of care, these models may serve as adjunctive tools to support clinical continuity when direct evaluation is not feasible. Nevertheless, the substantial variability in individual-level predictions underscores the need to use them alongside, rather than in place of, formal cognitive assessment.

## Data Availability

The raw data supporting the conclusions of this article will be made available by the authors upon request.
